# Enhancing Mechanical Properties and Flux of Nanofibre Membranes for Water Filtration

**DOI:** 10.3390/polym15153281

**Published:** 2023-08-02

**Authors:** Siddratul Sarah Binti Mohd Hami, Nor Dalila Nor Affandi, Liliana Indrie, Simona Tripa, Ahmad Mukifza Harun, Mohd Rozi Ahmad

**Affiliations:** 1 Textile Research Group, Faculty of Applied Sciences, Universiti Teknologi MARA, Shah Alam 40450, Selangor, Malaysia; siddratulsarah@gmail.com (S.S.B.M.H.); rozitex@uitm.edu.my (M.R.A.); 2 Department of Textiles, Leather and Industrial Management, Faculty of Energy Engineering and Industrial Management, University of Oradea, Universitatii Str. No. 1, 410087 Oradea, Romania; simona.tripa@didactic.uoradea.ro; 3 Nano Lab, Faculty Engineering, University Malaysia Sabah, Kota Kinabalu 88400, Sabah, Malaysia; mukifza@ums.edu.my

**Keywords:** nanofibres, electrospinning, water filtration, post-treatments, mechanical properties

## Abstract

Nanofibres have gained attention for their highly porous structure, narrow pore size, and high specific surface area. One of the most efficient techniques for producing nanofibres is electrospinning. These fibres are used in various fields, including water filtration. Although they possess the ability to filter various components, the fibres generally have low mechanical strength, which can mitigate their performance over time. To address this, studies have focused on enhancing nanofibre membrane strength for water filtration. Previous analyses show that the mechanical properties of nanofibre mats can be improved through solvent vapour treatment, thermal treatment, and chemical crosslinking. These treatments promote interfibre bonding, leading to the improvement of mechanical strength. However, excessive treatment alters nanofibre behaviour. Excessive heat exposure reduces interfibre bonding, while too much solvent vapour decreases pore size and mechanical strength. Thus, a comprehensive understanding of these post-treatments is crucial. This review examines post-treatments aiming to increase the mechanical strength of nanofibre mats, discussing their advantages and disadvantages. Understanding these treatments is essential for optimising nanofibre membrane performance in water filtration and other applications.

## 1. Introduction

**Figure 1:**
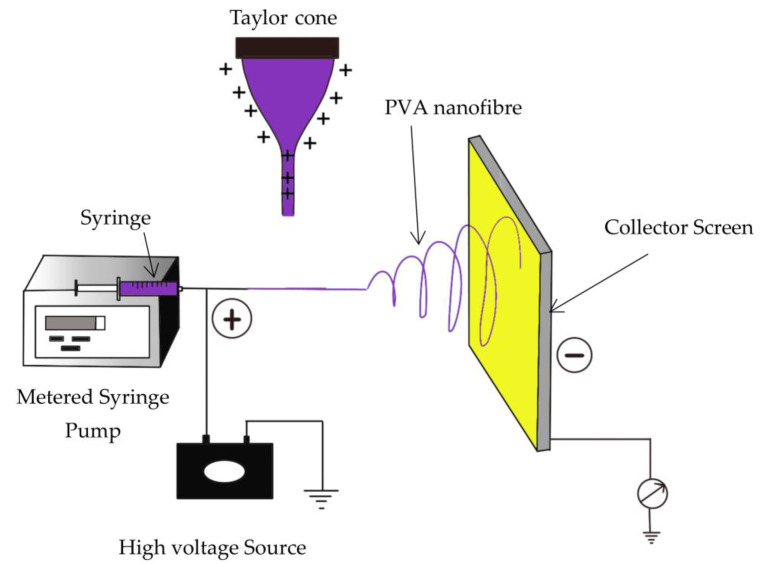
Schematic diagram of electrospinning.

**Figure 2:**
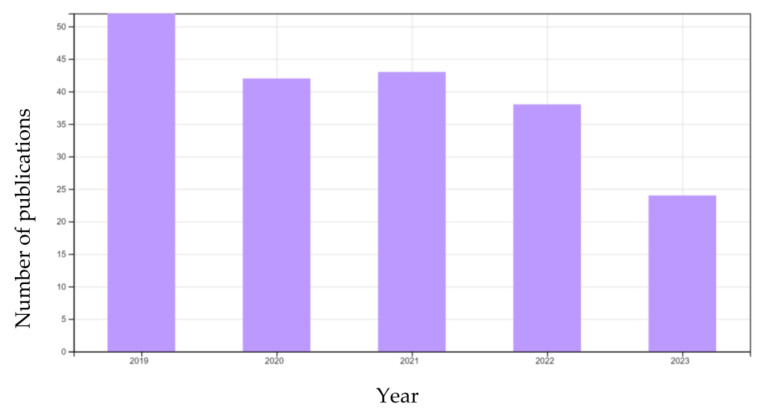
Graph showing the total relevant published articles on electrospun nanofibre-related research between 2019 and July 2023 by research outputs with the search topic “electrospun nanofibre water filtration” carried out using the Web of Science online search system as of 17 June 2023.

In the world of nanomaterials, nanofibres exhibit immense potential, characterised by their diameter ranging from tens of nanometres to less than 1 micrometre [1,2]. Their popularity is primarily from their remarkable surface area-to-volume ratio and high porosity, rendering them robust and appealing candidates for numerous advanced applications [1]. Additional attributes such as morphology, a high aspect ratio of length to diameter, and high molecular orientation along the fibre axis [3], can be configured and produced by manipulating the materials [1], changing the solution parameters, environmental variables, and employing electrospinning [4]. Most of these parameters are correlated methods to produce nanofibres including wet spinning, dry spinning, or melt spinning, template synthesis, solution blow spinning, and force spinning, but the most efficient and common way is through electrospinning [2]. Electrospinning involves the use of electrical charge to transform liquid polymer droplets into a continuous polymer nanofibre or nonwoven nanofibre structure [3]. This process entails charging the liquid polymer droplet with electricity, creating a jet, and subsequently subjecting it to stretching and elongation, resulting in the formation of a fibre (Figure 1) [5]. When the physical features and chemical versatility of nanofibres emerge, they can be used in a wide variety of applications, such as air and water purification, drug (gene) delivery, wound dressing, enzyme immobilisation, tissue engineering, gas storage, and sensor and electrode production in electronics [4]. The ongoing publishing of related electrospun nanofibres in water filtration indicates the rise and development of electrospinning technology within the field. Figure 2 shows a summary of all papers related to electrospun nanofibre in water filtration as a search subject between 2019 and June 2023. The Web of Science internet search technology provided assistance for the quantitative data in the literary study surveys.

Filtration of wastewater is required because industrial effluent usually pollutes water resources, causes environmental imbalances, and has a negative impact on human health [6]. Filtration can be defined as a process where solid substances are removed from a fluid or liquid wastewater by running them through a porous medium [7]. There are different filtration modes in water treatment, such as treatment of drinking water, treatment of wastewater, water disinfection [8], or dewatering [6]. In wastewater treatment, the use or potential use of electrospun nanofibres for water filtration has been gaining attention as the fibres have high porosity and good functional abilities [6]. There have been many studies on water filtration using nanofibre technology. Some studies focus on the use of nanofibre membranes for specific water treatment, such as the removal of oil, dyes, bacteria or viruses, or desalination [8,9,10,11]. Others have investigated the improvement of filtration applications by developing a novel polyvinyl fluoride (PVDF) nanofibrous multilayer membrane with additional lamination [9]. Liu et al. (2022) discuss one of the most recent developments of advanced functional nanofibres for wastewater treatment. Their research resolves an issue of poor adsorption/photocatalytic performances and low potential recycling. In resolving this issue, they produce large-area recyclable CFC/BiOBr/ZIF-67 filter-membrane-shaped photocatalyst prepared by in situ growth of BiOBr/ZIF-67 nanocomposites on carbon fibre cloth (CFC) [12]. A research article by Li et al. (2022) discusses a novel S-scheme heterojunction photocatalyst for deleterious pollutant removal. The membrane was developed by in situ growing Bi2WO6 nanosheets with oxygen vacancies (OVs) on TaON nanofibres. Furthermore, according to the systematic photoreaction test, the membrane has high practicality in eliminating pollutants in aquatic environments [13].

**Figure 3:**
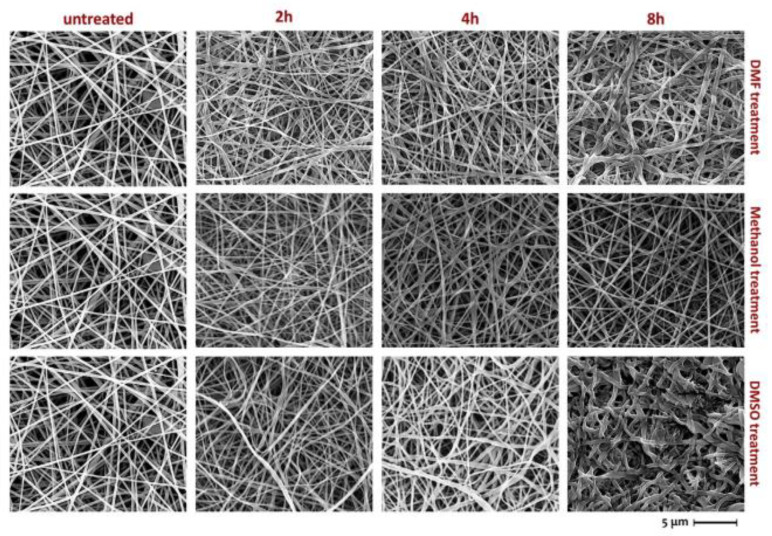
SEM images of nanofibre PVA before and after solvent vapour treatment with various solvents and times at a set temperature of 40 °C [25].

However, nanofibre membranes possess low mechanical strength due to their high porosity and weak bonding at the junction of fibres, small fibre diameters, and nonwoven structure [1]. Therefore, numerous research studies have focused on enhancing the mechanical strength and water flux of nanofibre membranes, as these pose significant challenges in the production of nanofibre membranes for water filtration. Several literature reviews have been conducted, exploring various aspects such as fabrication techniques and applications, parameters influencing the process, and materials used to produce electrospun nanofibres [14,15,16,17,18,19,20]. The reviews on the mechanical properties of nanofibres and water flux for water filtration are vital as these properties are the main hindrances in implementing nanofibre membrane use for water filtration. Achieving high mechanical strength is required for water filtration, as the membranes have to withstand operational conditions and handling during module fabrication [21]. Previous studies have explored different approaches, such as solvent vapour treatment, thermal treatment, and chemical crosslinking to improve the mechanical properties of nanofibre mats [21,22,23]. These treatments facilitate stronger interfibre bonding, thereby bolstering mechanical strength. Nevertheless, it is crucial to note that excessive treatment can adversely affect the behaviour of nanofibres. Excessive heat exposure, for instance, can weaken interfibre bonding, while an excessive amount of solvent vapour may lead to reduced pore size and diminished mechanical strength (Figure 3). Therefore, gaining a comprehensive understanding of these post-treatments is imperative. Additionally, a requirement for high permeate flux or anti-fouling properties is vital, as they can minimise the energy consumption involved in water filtration processes [24]. This review critically examines various post-treatments aimed at augmenting the mechanical strength of nanofibre mats, discussing their advantages and disadvantages. By comprehending these treatments, researchers can optimise the performance of nanofibre membranes not only in water filtration but also in other applications.

## 2. Nanofibre as Water Filtration Membrane

**Figure 4:**
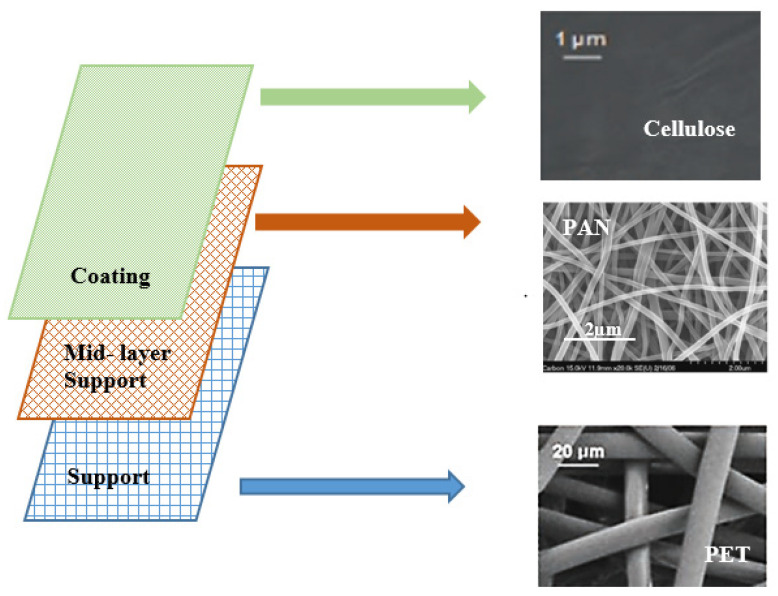
Hierarchical structure of cellulose-based TFNC ultrafiltration membrane. Figure was adapted and modified from [4].

Among the techniques for creating nanofibres, electrospinning is a special synthetic technique that employs electrostatic forces to create fibres with a diameter of 40 to 200 nm [26]. A high voltage supply source, a syringe to feed the polymer solution, a metal spinneret or a needle to transfer the polymer solution, a syringe pump to pump the polymer solution from the syringe reservoir, and a ground collector to collect the ejected polymer fibres are the five main parameters used in electrospinning [27]. By creating an electrical potential between a polymer droplet kept at the spinneret’s nozzle and the grounded collecting plate, fibre extrusion is formed [27]. Electrospinning is popular as it is a simple, versatile, and viable process for producing continuous nanofibres from synthetic and natural polymers [27]. Polymeric nanofibres were developed as filtration membranes because they offer advantages such as high porosity, low basis weight, high surface area, manageable pore size, and continuously interconnected pores [27]. These advantages have been utilised as water filtration media, which effectively remove contaminants [4] for microfiltration, ultrafiltration, and nanofiltration [28]. The nanofibres are usually layered on top of strong nonwoven polymers, which act as a support layer to increase the fibres’ mechanical properties [28]. The three-layer ultrafiltration membrane’s structure is depicted in Figure 4, with the bottom layer being made of polyethylene terephthalate (PET), the middle support being electrospun polyacrylonitrile (PAN) nanofibres, and the top layer being cellulose from an ionic liquid solution (barrier) [4].

## 3. Advantages and Challenges of Using Nanofibre Membranes in Water Treatment

### 3.1. Advantages

Electrospun nanofibres have a large surface-area-to-volume ratio, high porosity, excellent water permeability, and flexibility, which enables them to be widely applied in air filtration, water filtration, water treatment, desalination, and adsorption [2,4,20,29,30]. Electrospinning is also a practical and simple way to generate nanostructures as it is easy to control the nanofibre microstructure arrangement, diameter, and material selection [2]. The main advantage of using nanofibre membranes in water treatment is that the membrane has a large surface-area-to-volume ratio. Having this property, the electrospun nanofibres mat provides good toxin adsorption particularly in the case of viruses, dyes, and heavy metal ions among others [4]. Furthermore, electrospinning can increase mechanical strength without considerably changing the morphology and dimensions of the membranes [29,31]. Electrospinning can create nanofibres with unique shapes at a low cost and with a high rate of production [32]. The addition of nanofibres to a standard micron-sized fibrous filter medium can increase the separation efficiency from 61% to 91% [23].

### 3.2. Challenges

The main challenge relates to the fouling of the nanofibre membranes. Pore blockage often occurs resulting in high filtration resistance and degradation of filtration performance [22,33]. In addition, nanofibre membranes have weak mechanical strength due to their high porosity and weak bonding at the junction of the fibres [22,34]. The poor mechanical strength can also be attributed to its small fibre diameters and highly porous structure [29]. A consequence of the defect-prone nature of nanofibre mat layers is that it creates challenges for their application in filtration, such as wastewater treatment, where the nanofibre membranes are liable to suffer from shrinkage and distortion [33]. Another defect is scratching, whereby nanofibre membranes start to delaminate [22] when exposed to a high-pressure backwash. The insufficient mechanical strength and large pore size dispersion of nanofibre membranes are major setbacks to their use in membrane distillation [22].

## 4. Mechanism of Enhancing the Mechanical Properties of Nanofibre Membrane

**Figure 5:**
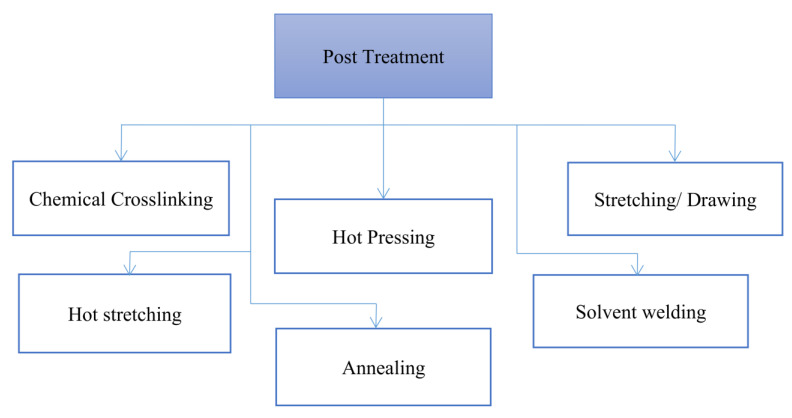
Mechanisms in enhancing nanofibre membrane.

Mechanical strength is defined broadly as the ability to withstand the stress of physical forces. To successfully commercialise a filtration process, the mechanical qualities of membrane systems must be improved [34]. To endure operational circumstances, pressure-driven membrane filtering technologies require appropriate mechanical strength [35]. In enhancing the mechanical strength of nanofibre membranes, it is crucial to understand the mechanism behind them. The first mechanism is crosslinking of fibre or interfibre bonding. Crosslinking involves chemically bonding the polymer chains within the nanofibre matrix, resulting in a stronger and more stable structure [22]. Treatments that use crosslinking mechanisms to enhance mechanical strength are chemical crosslinking, physical crosslinking, and enzymatic crosslinking. For instance, to chemically crosslink gelatine nanofibre, glutaraldehyde often uses as a crosslinking agent [36]. The mechanism of chemical crosslinking is in an earlier paragraph. In physical crosslinking, UV irradiation uses high energetic light to control the crosslinking density [37]. While enzymatic crosslinking is often performed by genipi or microbial transglutaminase to form covalent crosslinks between the primary amine residues [37]. Hybridisation with other materials also can efficiently enhance nanofibre membranes. The mechanical strength of nanofibre membranes can be increased by blending or adding other substances such as polymers, nanoparticles, or strengthening agents. These extra components can act as reinforcement, improve interfacial bonding, and overall boost membrane strength [38]. Another way is through structural modifications. It involves modifying the nanofibre structure, such as altering the fibre diameter and aspect ratio or introducing hierarchical structures. For instance, core-shell nanofibre geometry improves the mechanical properties of nanofibre membranes and has been thus introduced in industrial wastewater treatment [39]. The last way is through post-treatment, which includes exposure to solvent vapour [22,25,31,40], chemical crosslinking [21,41], hot pressing, annealing [23,42,43,44,45,46,47], hot stretching, and stretching/drawing (Figure 5).

**Figure 6:**
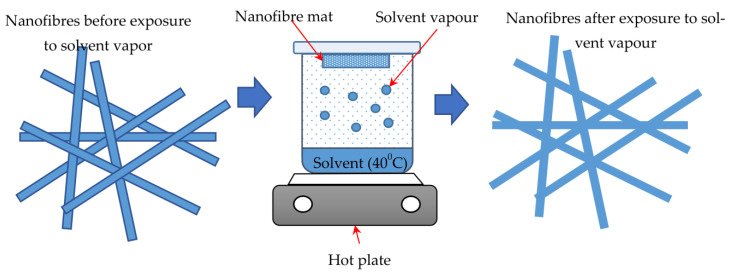
Schematic illustration of solvent vapour process. (Adapted and modified from [31,40]).

Solvent vapour is one of the most preferable methods to treat nanofibre membranes. With this technique, the nanoscale phase separation is more organised, and the active layer will have a better thermodynamic stable morphology [22,40]. This treatment also promotes the physical fusion between the fibres at their junctions and gives a smoother surface, which controls the fouling rate and hence increases the nanofibre membrane strength [22]. The treatment involving solvent vapour is applied for different reasons, such as to generate attoliter reactors, weld nanofibre mats into conductive films, introduce secondary nanostructure into electrospun fibres, and infiltrate the channels in a porous template with polymeric materials [31]. As reported by Rianjanu et al. (2018), the nanofibre mat is cut into sizable coupons and placed on a petri dish, which is then placed on top of the beaker with the coupons on the inside face of the beaker. The amount of solution used varied according to the requirements [25]. During this process, nanofibrous samples are directly exposed to solvent vapours to fuse fibres at their junction points [48]. The schematic diagram of the solvent vapour process is shown in Figure 6. In another controlled experiment, a nanofibre mat was exposed to solvent under partial pressure in order to fuse electrospun nanofibres made of both semi-crystalline and amorphous polymers [31].

**Figure 7:**
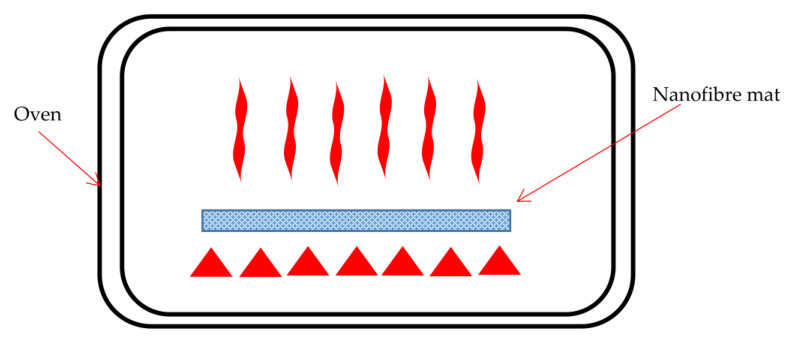
Schematic illustration of thermal treatment of nanofibres. (Adapted and modified from [49]).

Heat-treating the membranes, as shown in Figure 7 [49], is another method for enhancing the mechanical properties of electrospun fibrous membranes. Heat-treatment or annealing of these membranes/fibrous webs promotes crystallinity because higher temperatures cause the rearrangement of polymer chains [49,50]. The use of harmful chemicals can be eliminated, making it a straightforward and environmentally beneficial procedure [51]. Additionally, partial fibre fusion can take place, resulting in interfibre welding at the locations of the fibre crossovers, giving these membranes structural integrity [49,52]. For optimal results and to prevent completely melting the fibrous membrane, it was suggested that annealing be conducted at temperatures above those needed for crystallisation and below the melting point of the polymer [49]. By reordering molecular chains in amorphous regions and interfibre fusion due to the partial melting of fibres at junction points, heat-treatment in these conditions enhances crystallinity [49]. These two procedures frequently enhance the mechanical properties of electrospun fibrous membranes [49,53]. For the construction of an ideal composite structure, a proper combination of heating rate, temperature, and time for the thermal treatment is crucial, ensuring superior cycle performance and a higher rate of discharge capability [54]. According to certain research, heat-treatment may reduce the membrane’s pore size and fibre diameter [50].

**Figure 8:**
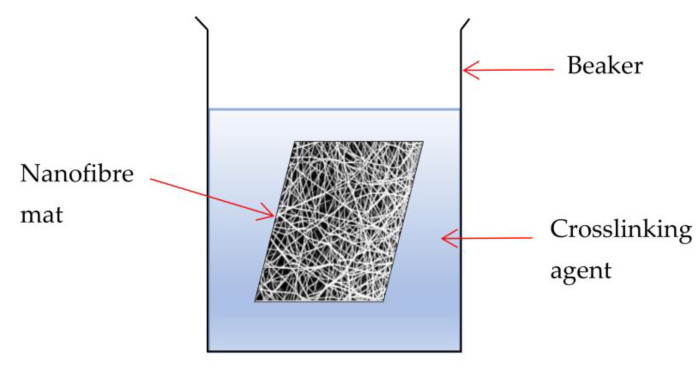
Schematic illustration of chemical crosslinking on a nanofibre mat. The figure was adapted and modified from [41].

The fibre–fibre connection can also be strengthened chemically through the use of cross-linking [21]. Hydrophilic electrospun nanofibre membranes commonly use this strategy to increase their water integrity [21]. Covalent bonds are generated between the molecular chains of a polymer through chemical crosslinking, increasing its molecular weight and improving mechanical qualities, such as strength, stiffness, abrasion resistance, hardness, and thermal stability, among others [49]. The chemical structure of the reactants involved, as well as their concentration, determines the kinetics of a crosslinking reaction [39,49]. As a result, the crosslink density and rate of reaction can be altered by adjusting the crosslinker concentration and reaction conditions [50]. Another research study states that chemical measurement can improve bonding at junction points in the nanofibre membrane by welding or soldering the fibres together, as well as changing the degree of molecular orientation of the polymer, which is thought to have a significant impact on the nanofibre’s mechanical properties [41]. The chemical crosslinking on a nanofibre mat is illustrated in Figure 8. Using a proper crosslinker, the crosslinking procedures can be established by the interfibre linkages in electrospun membranes [49]. Methods to improve the mechanical properties of electrospun nanofibres have been well-reported in previous studies. However, the effectiveness of these methods to enhance the mechanical properties and flux of the electrospun membrane is not yet understood. Hence, this review paper highlights the methods for improving mechanical strength, such as solvent vapour treatment, heat-treatment, and chemical crosslinking. This review also highlights the effect of these treatments on water flux, which is vital in water filtration. By increasing the mechanical strength and water flux, the nanofibre membrane can then be applied to improve the water filtration process in the future.

## 5. Mechanical Properties and Water Flux of Nanofibre Membrane after Solvent Vapour Treatment

### 5.1. The Mechanical Properties of the Nanofibre Membrane after Solvent Vapour Treatment

According to Abd Halim et al. (2019), formic acid was used to conduct the solvent vapour technique. The electrospun Nylon 6,6 was treated at different time intervals of 5, 12, 24, and 48 h at room temperature [22]. It was reported that the tensile strength of the nylon 6,6 nanofibre mat after treatment increased with greater exposure time. The tensile strength increased by 91% after 24 h exposure compared to untreated samples. This increase in mechanical strength was caused by the condensation of formic acid. On the other hand, the incremental change in tensile strength with increased exposure time was due to the higher number of fibre fusions between the fibre junctions. In addition, the solvent vapour treatment promoted random fusing across the nanofibre mat. Random fusing resulted in a greater tensile strength distribution, therefore, boosting the nanofibre mat’s tensile strength. It also expanded the fibres by combining them at different locations. This correlates with FESEM images of the untreated membrane with the treated membranes of different exposure times. In addition, overexposure to solvent vapour damages the nanofibre mat, thereby lowering its mechanical strength. The tensile strength diminished when the exposure duration was set to 48 h. Uncontrolled swelling caused fusion between fibre layers as a result of excessive exposure to solvent vapour. Consequently, the tensile strength distribution among fibres was narrow.

A study conducted by Huang and his colleagues used two different methods for solvent vapour treatment. In method A, the nanofibres were left on an aluminium foil, while in method B, the nanofibres were removed from the foil. Two types of polymeric electrospun membranes were tested, including electrospun Polyacrylonitrile (PAN) and Polysulfone (PSU), both using N, N-Dimethylformamide (DMF) for the solvent treatment [29]. The study found the tensile strength and modulus of PAN increased using method A. However, with method B, there was only a small increase in both tensile strength and Young’s modulus (56% and 25%, respectively). The results for the PSU membrane corresponded with the results for the PAN membrane, where both tensile strength and Young’s modulus increased by 400%. In method B, the tensile strength and Young’s modulus of the membranes increased by 80% and 110%, respectively. The mechanical property results are consistent with the SEM data, indicating that solvent-induced fusion at the fibre junction areas was responsible for the abrupt rise in modulus and tensile properties in PAN and PSU membranes. In other words, the post-treatment condensation of DMF vapour kept the electrospun mat relatively “wet”, resulting in a fusion between the interfibre connections. The swelling of the fibres may also aid in increasing the strength of the membranes. Method B-treated membranes are projected to have less fusion and swelling; therefore, the mechanical properties are unlikely to increase significantly. It is also worth noting that the swelling effect may diminish when the electrospun nanofibre membranes (ENMs) are submerged because DMF diffuses out of the fibres while this does not occur with fibre–fibre fusion. The use of solvent vapour to treat electrospun membranes was also investigated in other studies [25,31,41,48,49]. The solvents used in these studies varied from formic acid, acetone, methanol, N,N dimethylformamide (DMF), dichloromethane (DCM) dimethyl sulfoxide (DMSO), and di-methylacetamide (DMAc). The studies showed improvements in the tensile strength of treated nanofibre membranes. Furthermore, this improvement is related to exposure time, which increases as the tensile strength increases [25,31,41,48,49]. This correlates to an increase in fibre fusion between fibre junction points in both the in-plane and normal-to-plane directions, preventing mat de-lamination [25,31,41,48,49]. However, overexposure to solvent vapour tends to damage the fibre structure, thus decreasing the tensile strength and at a certain point, the membrane becomes slightly transparent [25]. This is due to excessive fibre fusion [22,25]. Therefore, the determination of exposure time is critical in improving the tensile strength, while preventing damage to the nanofibre membrane.

To sum up, the application of solvent vapour treatment has been shown to enhance the tensile strength of nanofibre membranes by fusing the fibre joints. Consequently, these nanofibre membranes hold promise for application in the water filtration industry, as the treatment can mitigate issues such as delamination, shrinkage, and distortion when subjected to high-pressure backwashing. However, these past research studies have not extensively addressed the lifespan of the membrane after it has been treated with solvent vapour. Hence, conducting a study on the membrane lifespan is highly recommended to gain insights into the potential degradation or structural alterations of the membrane over time, which can be attributed to environmental factors such as moisture, temperature, or chemical exposure. In addition, the use of solvent vapour may pose health hazards to humans due to the inhalation of chemicals, warranting further investigation in future studies. From the authors’ point of view, it is strongly advised to prioritise the utilisation of environmentally friendly chemicals, as they offer both human and environmental benefits.

### 5.2. The Effects of Solvent Vapour Treatment on Water Flux

High permeate flux and anti-fouling are important because they can lower energy requirements for water filtration [27]. Therefore, it is essential to be able to access the filtration properties, such as water permeability, of the nanofibre membrane. In a study by Abd. Halim et al. (2018) on the effects of solvent vapour treatment on electrospun nylon 6,6 nanofibre membrane, the electrospun membranes were exposed to formic acid vapour for various durations (5, 12, 24, and 48 h) at room temperature. The results showed that the steady-state pure water permeability of the membrane treated for 5 h was 1% lower than that of the untreated membrane. However, if the membrane was treated for 48 h, the water permeability decreased by 44% compared to that of the untreated membrane. This relates to the effect of pore size on the exposure time. As the treatment time increases, the pore size decreases, hence a decrease in water permeability. To induce fusion fibres at contact locations, a shorter period of exposure is preferred. Only a small portion of the nanofibre surfaces dissolve, just enough to create physical crosslinks between the fibres. Membrane porosity and permeability will not be affected as a result of this, and membrane fibres will be strengthened through crosslinking. Therefore, the membrane should be exposed for 5 h to prevent a reduction in porosity and water permeability [22].

Liu et al. (2016) studied the solvent vapour treatment of cellulose acetate and polyvinylidene fluoride (CA/PVDF) electrospun nanofibre membranes. The results show the M1 membrane (untreated) had the highest water flux, at 17.4 m^3^/m^2^·h. In comparison, the M5 membrane (membrane treated with acetone at 500 °C for 10 min) had a flux of 0.12 m^3^/m^2^·h which was lower than the M1. In other words, the water flux for M5 was reduced by 0.7%. The filtration rate of M5 (99.99%) was much higher than that of M1 (44.4%). This is due to the smaller pore diameter of M5 (pore diameter: 0.74 micrometres) than M1 (pore diameter: 1.93 micrometres) [30].

Overall, the solvent vapour treatment causes the water permeability of the nanofibre membrane to decrease slightly. This concurs with the findings of other studies [8,22,25,55] where exposure to solvent vapour has been shown to cause a decrease in pore size, which reduces the porosity and permeability [20]. Others claim that due to better fibre bonding caused by solvent vapour treatment, the electrospun nanofibre membranes (ENMs) increase their resistance to compression and, therefore, permeability drops [8,22,25,55]. Even though solvent vapour treatment reduces permeability, it also reduces fouling, which extends the membrane life and lowers operating expenses. In general, solvent vapour treatment has little effect on ENMs’ flux performance, particularly when evaluated at greater hydraulic pressures. In addition, it results in a significant increase in membrane integrity without sacrificing the pore size and porosity of the resultant membranes, which is a desired property for filtration applications. Based on the comprehensive information provided by all these reviews, the present study advocates for the increased consideration of solvent vapour application in the electrospun nanofibre membrane (ENM). Subsequent research studies may focus on exploring the utilisation of non-hazardous chemicals for solvent treatment techniques.

## 6. Mechanical Properties and Water Flux of Nanofibre Membrane after Heat-Treatment

### 6.1. The Mechanical Properties of Nanofibre Membranes after Heat-Treatment

Heat-treatment is commonly applied to electrospun membranes. Wang et al. (2019) heat-treated Polyphenylsufone (PPSU) electrospun nanofibre membranes (ENMs) in an oven at 240 °C for 1 h [42]. To prevent shrinkage, the sidewalls of the membranes were sealed during the process. After treatment, the heat-treated membranes had dramatically improved tensile characteristics as compared to untreated membranes. The elastic modulus and the breaking stress increased, respectively, to 930% and 853% of that of the untreated membranes. The loose structure and poor cohesiveness of the untreated PPSU membrane were due to the poor fibre-to-fibre bonding in the membrane. When the untreated PPSU membrane underwent the heat-treatment process, the polymeric fibres fused at the intersections during the heating process to generate a more compact and integrated structure. Furthermore, during the heat-treatment, the segmental movements of the polymer chains effectively eliminated the internal stress created during the spinning process, resulting in improved structural stability. In addition, by anchoring the sidewalls of the membranes, segmental motions were able to rearrange the polymer chains along the direction of the external force applied, increasing the orientation degree of the amorphous polymer. All these factors aided in improving the structural stability and mechanical qualities of the membrane [42].

In a study conducted by Himaeigohal et al. (2012), the blended PSU/PAN membrane was heat-treated for 5 h at 185 °C. The results show that a heat-treated membrane can withstand substantially higher levels of stress than an untreated membrane. The treated membrane could withstand 2.507 N/mm^2^, whereas the untreated membrane could only tolerate 1.266 N/mm^2^ until it ruptured. This shows a difference of 198% in tensile strength. Heat-treated membranes, on the other hand, had a lower strain level (5.927%) than untreated membranes (7.854%). These findings show that heat-treatment improves membrane durability. Lower strain indicates less plasticity and a more rigid structure. In addition, untreated membranes scattered and tore during wastewater filtering, but heat-treated membranes remained intact. As a result of the heat-treatment, the mechanical qualities were improved [47].

Heat-treatment has been investigated in other studies [23,43,44,45,46,47]. Although it increased tensile strength in all cases [23,43,44,45,46,47], it also resulted in a decrease in elongation at break [43,44,45,46,47]. The tensile strength was increased due to the fibres fusing with one another. Moreover, the thickened fibres and a greater degree of crystallinity enhanced the stiffness of the single fibres, while decreasing their ductility. Therefore, thermal treatment can increase tensile strength, while also affecting the stiffness or elongation at break.

### 6.2. The Effects of Heat-Treatment on Water Flux

Heat-treatment has a great impact on the water permeability of electrospun nanofibre membranes (ENMs). According to Ozbey-Unal et al. (2020), it also produces an increase in permeate flux and the salt rejection of polyvinyl fluoride (PVDF) ENMs. In this study, the treatment was for 1 h at a temperature of 100 °C. After heat-treatment, the permeate flux and salt rejection values increased from 26.4 kg/m^2^/h to 30.4 kg/m^2^/h and from 94.9% to 95.5%, respectively. The water flux was 115% higher than that of the untreated water. When the heat-treatment was conducted, the thickness of the membrane was reduced from 196.2 ± 9.8 µm to 188.4 ± 6.2 µm, resulting in a shorter total vapour transfer distance and a reduction in mass transfer resistance [44].

Another study shows that heat-treatment increased the membrane’s structural integrity and mechanical strength. This heat-treatment also helps to increase the flux of pure water [43]. In this study, polyphenyl sulfone (PPSU) electrospun nanofibre membranes (ENMs) were treated for 1 h at 245 °C. The heat-treatment increased the structural integrity and mechanical strength of the membrane. As demonstrated by SEM pictures, the membrane had less compaction and higher porosity. The heat-treated membranes exhibited a bigger pore radius compared to that of the untreated one, resulting in a higher pure water flux of 144%. The rejection values for both treated and untreated membranes were nearly identical. The untreated and heat-treated membranes both obtained 100% turbidity rejection, implying that the wastewater-suspended particles were properly removed.

Conversely, other studies show a decrease in water flux after heat-treatment of the ENMs [43,44,45,47]. Li et al. (2013) state that heat-treatment causes the pore size and porosity to decrease, causing a lower initial flux compared to that of the untreated ENMs. They also state that the pores of the heat-treated membranes were more resistant to liquid flow than the pores of the untreated membranes and were more easily blocked by the filtered particles due to their smaller pore size and porosity. As a result, it leads to the formation of a filter cake and increased the particle rejection. These results were observed in another study [45] where the heat-treated membrane also showed a considerably superior retention efficiency for the colloidal nanoparticles, despite having a lower permeability due to the dense cake layer development. This was considered due to the propensity for disintegration at the maximum applied feed pressure, i.e., 2 bar.

Given all these reviews, water flux is highly impacted by changes to pore size and porosity caused by heat-treatment. If pore size and porosity increase, the water flux also increases. However, the optimal temperature for treating electrospun nanofibre membranes is scarcely reported by previous studies. Therefore, it is recommended to optimise the temperature for heating electrospun nanofibre membranes, as this critical factor significantly influences the resulting pore size and porosity of the membranes.

## 7. Mechanical Properties and Water Flux of Nanofibre Membrane after Chemical Crosslinking

### 7.1. The Mechanical Properties of Nanofibre Membranes after Chemical Crosslinking

A study shows that polydopamine (PDA) modification improves the mechanical characteristics of both polyacrylonitrile (PAN) and polysufone (PSu) membranes [21]. The electrospun nanofibre membranes (ENMs) were coated with PDA and tris-HCl with treatment periods of 1 and 18 h at room temperature. The results show that PAN ENMs had a 100% increase in tensile strength and Young’s modulus following alteration, signifying a stronger and stiffer material. In addition, the ultimate tensile strength and Young’s modulus enhancement for PSu ENMs were found to be 80% and 210%, respectively. However, for both PAN and PSu, there was no statistically significant change in elongation at break following alteration, indicating that nanofibre flexibility was preserved. The PDA layer binds nanofibres together, promoting interconnectivity within the mat. Furthermore, the PDA-coated electrospun nanofibre membranes (ENMs) demonstrated increased mechanical characteristics. The fibres were glued together at places of overlap whereupon the PDA layer formed in the space between them. When comparing the mechanical qualities of PAN and PSu after 18 h of modification against 1 h of coating time, there was essentially no improvement in the mechanical properties. A mechanical strength test demonstrates that extending the coating period does not improve the strength and is, therefore, less cost-effective.

Cai et al. (2016) conducted a study using different ratios of ethanol/acetone for the post-treatment of bamboo cellulose (B-CA) electrospun nanofibre membranes (ENMs). The findings indicate that the strength and modulus of the treated B-CA membranes with 95/5 (*v*/*v*) ethanol/acetone mixture solutions increased dramatically to 203% that of the untreated sample. Based on this mechanical property data, the increase in tensile properties and modulus in the B-CA membranes can be attributed to an increase in fibre density as well as solvent-induced fusion at the fibre junctions [41].

Irradiation is another way to increase the mechanical properties of nanofibres by crosslinking them. It is a green approach to the construction of hydrogels or scaffolds and is mostly used to sterilise biomedical products [56]. In electron beam (EB) irradiation, a sample is blasted with high-energy electrons, resulting in a cascade of these electrons moving through the target material. High-energy electrons are used as the radiation source in the EB approach [57]. The electrons are focused onto a scan horn of a particular size and scanned in a sweeping motion to create an electron curtain [57]. Following that, the product is transported at a precise and regulated pace past the scan curtain. This method has received a lot of consideration for routine medical product sterilisation [58].

Wongkrongsak et al. (2022) conducted research on the electron beam (EB)-induced cross-linking of silk fibroin-poly (ethylene oxide) SF-PEO nonwoven nanofibres prepared through additive-free electrospinning in a pure water system. A low-energy electron beam accelerator (with an energy of 145 keV and a current of 1.1 mA was used to perform electron beam (EB) irradiation. The samples were put on the irradiation tray and exposed to radiation in atmospheres of air and N2 at standard pressure and temperature. To form the nonwoven nanofibres for samples SF-PEO-0, SF-PEO-10, SF-PEO-25, SF-PEO-50, SF-PEO-75, and SF-PEO-100, each sample was exposed to radiation at various absorbed doses of 0, 10, 25, 50, 75, and 100 kGy, respectively. The mechanical behaviour of the SF-PEO nanofibres increased significantly after EB irradiation. The tensile strength of SF-PEO after irradiation doses of 10 and 24 kGy increased to 142.17% and 266.27% of that of untreated SF-PEO. The Young’s modulus of SF-PEO following irradiation doses of 10 and 24 kGy also showed an increase of 131.61% and 58.26%, respectively. The increase in mechanical strength was due to the partial crosslinked network established in the nanofibres, which acts as reinforcement inside the nanofibre matrix and the entangled network of the nanofibre mat [56].

A study on the influence of the electron beam on the poly L-lactide acid/carboxy-methyl starch/β-tricalcium phosphate (PLLA/CMS/β-TCP) composite nanofibres was conducted by Yusof et al. (2020). To reduce the heat produced by the electron beam, composite nanofibres were exposed to radiation at dosages of 5, 30, and 100 kGy (EPS 3000, Nissin High Voltage, Chiyoda-ku, Tokyo, Japan). The setpoints for the voltage and current were 2 MeV and 1 mA, respectively. In this study, the EB decreased the mechanical strength of the nanofibres. With higher irradiation doses, a drop in tensile strength was observed. Following irradiation at 100 kGy, the tensile strength decreased by 61.92%. This could be connected to the irradiated composite nanofibres’ polymer chain breakdown. The polymer chain’s rearrangement to a crystallite structure from the broken chain contributed to the brittle behaviour during the tensile test and reduced plasticity of the nanofibres [57].

Chemical crosslinking can increase the mechanical strength of nanofibres without affecting their flexibility. The improvement of mechanical strength is due to the fusion of fibres via solvent treatment. The solvent causes the fibres to become relatively wet and swollen. Determining the soaking time is crucial: after the optimal period of soaking, no improvement in mechanical strength is observed. Although crosslinking using EB irradiation has not been used in water filter applications, it is worth noting that EB irradiation can transform water-soluble nanofibres into insoluble ones. This is carried out by initiating crosslinking through the free radical reaction pathway of the nanofibres [58].

### 7.2. The Effects of Chemical Crosslinking on Water Flux

Although chemical crosslinking increases the mechanical properties of electrospun nanofibre membranes (ENMs), it reduces their water permeability. Huang et al. (2014) state that with increased pressure, the pure water permeability of all three membranes (PAN pre-wet, PAN dry, and PAN–PDA 1 h ENMs) declined due to the resultant compaction of the membrane. The permeability of the PAN–PDA 1 h ENMs was not enhanced compared to that of the dry, unmodified PAN ENMs. In fact, there was a modest drop in permeability (down to 96%), which is likely attributable to the coated samples’ reduced pore volume and pore widths. Nonetheless, for the PAN–PDA 1 h ENMs, the pore shape had a minor impact on the water flux performance, and the ENMs retained their high permeability. The pure water permeability of all three membranes (pre-wet PSu, dry PSu, and PSu–PDA 1 h ENMs) declined with increasing applied pressure, similar to that of the PAN ENMs. The PSu–PDA 1 h ENMs, such as the pre-wet PSu ENMs, had a greater flux than that of the dry PSu ENMs. When compared to dry PSu ENMs, the flux of the PSU-PDA 1 h increased by 118%. This is because the PDA alteration improves surface hydrophilicity, while also potentially blocking smaller pores. The improved surface wettability of the PSu–PDA 1 h ENMs overcomes the effect of reduced pore volume and pore diameters. The flux performance of the PSu–PDA 1 h ENMs was similar to that of the pre-wet PSu ENMs [29].

Cai et al. (2016) concur with Huang et al. (2014) who found that, after solvent-soaking treatments, the pure water permeability of B-CA ENMs dropped somewhat under the same applied pressure. Compared to the untreated membrane, the treated membrane decreased by 89.9%. This could be because the solvent-soaking procedure caused fibre cross-bonding and fibre deformation in the ENM, which reduced porosity and porosity interconnectivity (raising tortuosity) by clogging pores. The pore size was also lowered as a result of the blockage. The solvent-soaking treatment did not significantly reduce the B-CA ENMs’ pure water permeability while dramatically increasing their mechanical strength, making them ideal for water filtering applications. Altogether, chemical crosslinking reduces water permeability as it reduces pore size and porosity, although it can improve surface wettability and membrane mechanical strength [41].

## 8. Experimental and Computational Analysis of the Modifications of Nanofibre Membrane Strength

Recent advances in experimental and computational development have become potent tools for exploring and engineering the modifications needed to increase the strength of nanofibre membranes. Khatri et al. (2023) focus on enhancing nanofibre membranes for membrane distillation (MD) through nanofibre composite structure [36]. A sufficient tensile strength has to be constructed and attached to membrane modules as operational pressures are substantially lower than those for RO, UF, and MF [59]. Domaschke et al. (2018) use a 3D computational model of electrospun networks and their application to inform a reduced modelling approach. Through this 3D modelling, they found the relation between topology, micromechanical deformation mechanisms and macroscopic mechanical behaviour in electrospun nanofibre. The model can also take into consideration the interactions between fibres in terms of both actual material cross-links and contact formed during network deformation [60]. Chavoshnejad et al. (2021) conducted a different work in which they provide a brand-new computational model to forecast the mechanical behaviour of an electrospun fibre. This may be determined by considering its microstructure and the proportion of bonded cross-points. They analysed three cases with different percentages of bonding under uniaxial and biaxial tension. The study demonstrated that a linear increase in the single fibre’s elastic modulus increases the mat’s stiffness. In contrast, there is a nonlinear and parabolic relationship between the diameter of a single fibre and the stiffness of the mat. In order to control how stiff the mat is, the diameter of the fibres acts as the primary determining element [61]. Yin and Xiong (2018) conducted similar research, studying the mechanical behaviour of electrospun nanofibre membranes under biaxial tension. They used two macroscopic continuous finite element (FE) models with a uniform or directed nanofibre distribution. Due to the upward force along the long direction of the fibrous membrane, the modulus of the nanofibrous membrane decreases as the aspect ratio rises. Contrarily, the modulus increase as the aspect ratio increases, while the modulus of nanofibrous mats decreases as a result of the upward stress along the long direction of the fibrous mats. Therefore, future research on the bonding effect using bent fibres would be interesting [62].

## 9. Summary and Future Perspective

To summarise, all the post-treatments stated above were able to improve the mechanical characteristics of nanofibre membranes by random fusing across the membranes, with the vapour condensation during the post-treatment resulting in a fusion between interfibre contacts in the mats. The summary of findings regarding mechanical strength after post-treatment is shown in Table 1. The post-treatments increased the tensile strength of the nanofibre mats. This is highly advantageous as the fibres can withstand a higher load. Post-treatment also increases the fibre interbonding within the mat. However, if the mat is treated excessively, the nanofibres will behave differently depending on the type of post-treatment. Hence, it can be problematic if the post-treatment parameters are not carefully determined. For example, excessive solvent vapour exposure weakens the nanofibre mat’s mechanical strength. In addition, the water flux of the nanofibre mat is reduced due to the decrease in the pore size. Furthermore, the membrane’s porosity drops due to excessive exposure to solvent. As a result, the water flux of the membrane decreases during water filtration. These findings are compatible with those from other post-treatments, such as heat-treatment and chemical cross-linking. When comparing all three post-treatments, the solvent-soaking treatment caused a slight reduction in water permeability, while instilling great mechanical strength. Identifying the best parameters for each post-treatment step is therefore vital.

Yet, there are additional concerns regarding the post-treatments that necessitate attention from both researchers and manufacturers. One notable issue is the use of hazardous solvent vapours emitted during the treatment of nanofibre membranes. The authors firmly believe that giving priority to the use of environmentally friendly chemicals is of utmost significance, considering the well-being of humans, the environment, and other living organisms.

Addressing the challenges that may be faced by future research on optimising nanofibre membranes is crucial for advancing this field. The commercial manufacturing of high-quality nanofibres is still constrained despite the introduction of novel nanofibre synthesis processes. Particularly, larger-scale reproduction of the precise locations and orientation of nanofibres should be possible using industrial manufacturing processes [63]. Achieving consistent and uniform nanofibre morphology throughout the membrane is a challenge [64]. Dope preparation and electrospinning parameters influence fibre diameter, alignment, and distribution. Maintaining reproducibility across different batches is crucial for reliable performance. Electrospun nanofibre membranes can be susceptible to degradation or structural changes over time due to environmental factors, such as moisture, temperature, or chemical exposure. Ensuring long-term stability and performance retention is a challenge, particularly for applications requiring prolonged usage or exposure to harsh conditions [65]. Moreover, the shelf-life of electrospun nanofibre membrane after restoration is notably limited, and there has been insufficient research in this area. The electrospun nanofibre membranes must possess appropriate mechanical properties and durability to withstand various internal and external applications, including filtration, biomedical applications, protective clothing etc. Hence, a comprehensive study on the lifespan of electrospun nanofibre membranes is highly recommended in order to obtain durable membranes.

Discussion on industrial applications of nanofibre-based material made by Fadil et al. (2020), emphasises global companies on the commercialisation of nanofibre products in various sectors; air filters, wound dressing and technical textiles. The significant market growth in these products leads to their popularity. According to PR Newswire, the global market of nanofibres, valued at USD 927 million in 2018, is anticipated to increase at a compound annual growth rate (CAGR) of 36.2% to reach USD 4.3 billion by 2023. The report further analyses the market in prominent nations such as the USA, Canada, China, Japan, Europe, North America, and Asia across several end-use application categories, including packaging, automotive, electronics and semiconductors, aerospace, coatings, and energy. In addition, Global Industry Analysts Inc. provides comprehensive insights into the trends of the nanofibre markets in its report titled “Nanofibres: A Global Strategic Business Report”. The report stated that the Asia-Pacific region is the largest and fastest-growing market worldwide, attributed to significant investments in nanotechnology by both public and private sectors. This expansion is fuelled by the high demand for nanofibres in applications for performance apparel, taking advantage of their distinctive qualities such as high surface area, small fibre diameter, filtration abilities, thinness, permeability, and low basis weight, resulting in outstanding product functionality [64].

## Figures and Tables

**Table 1:** The mechanical properties of nanofibre membranes post-treatment.

Nanofibre Polymer	Treatment	Tensile Strength (MPa)	Young’s Modulus (MPa)	Ref.
Nylon 6,6	Solvent Vapour	Untreated: 737 MPa Treated: 1407 MPa	NA	[22]
Polyacrylonitrile (PAN) and Polysulfone (PSu)	Solvent Vapour	Untreated PAN: 5.59 MPa Untreated PSu: 0.83 MPa Method A (Nanofibres were left on an aluminium foil): PAN: 23.84 Mpa Psu: 3.35 MPa	Untreated PAN: 57.31 MPa Untreated PSu: 11.7 MPa Method A (Nanofibres were left on an aluminium foil): PAN: 523 Mpa Psu: 44.62 MPa	[29]
		Method B (Nanofibres were removed from the foil): PAN: 8.78 Mpa Psu: 1.47 MPa	Method B (Nanofibres were removed from the foil): PAN: 73.7 MPa Psu: 24.75 MPa	
Polyphenylsulfone (PPSU)	Thermal treatment		Untreated: 0.43 MPa Treated: 4.10 MPa	[42]
Polysulfone (PSU) & Polyacrylonitrile (PAN)	Thermal treatment	Untreated: 0.58 MPa Treated: 0.88 MPa	NA	[47]
Polyacrylonitrile (PAN) and polysulfone (PSu)	Chemical crosslinking	Untreated PAN: 5.27 MPa Treated PAN: 10.48 MPa	Untreated PAN: 73.1 MPa Treated PAN: 163.3 MPa	[21]
		Untreated Psu: 1.02 MPa Treated PSu: 2.18 MPa	Untreated Psu: 12.2 MPa Treated PSu: 38.3 MPa	
Bamboo cellulose (B-CA)	Chemical crosslinking	Untreated: 3.9 MPa Treated: 7.7 MPa	Untreated: 222.6 MPa Treated: 291.8 MPa	[41]
Silk-Fibroin/Poly(ethylene oxide)	EB irradiation	SF-PEO-10/N_2_: 142.17	SF-PEO-10/N_2_: 131.61	[56]
		SF-PEO-25/N_2_: * 266.27%	SF-PEO-25/N_2:_ * 58.26%	

* The percentage difference is between treated and untreated samples.

## Data Availability

Not applicable.

## References

[1] Lim C.T. (2017). Nanofiber technology: Current status and emerging developments. J. Prog. Polym. Sci..

[2] Toriello M., Afsari M., Shon H., Tijing L. (2020). Progress on the fabrication and application of Electrospun Nanofiber composites. J. Membr..

[3] Jiang S., Chen Y., Duan G., Mei C., Greiner A., Agarwal S. (2018). Electrospun nanofiber reinforced composites: A review. J. Polym. Chem..

[4] Ma H., Hsiao B.S. (2018). Current advances on Nanofiber membranes for water purification applications. Filtering Media by Electrospinning.

[5] Xue J., Wu T., Dai Y., Xia Y. (2019). Electrospinning and Electrospun Nanofibers: Methods, Materials, and Applications. J. Chem. Rev..

[6] Cui J., Li F., Wang Y., Zhang Q., Ma W., Huang C. (2020). Electrospun nanofiber membranes for wastewater treatment applications. J. Sep. Purif. Technol..

[7] Eyvaz M., Arslan S., Gürbulak E., Yüksel E. (2017). Textile Materials in Liquid Filtration Practices: Current Status and Perspectives in Water and Wastewater Treatment. Textiles for Advanced Applications.

[8] Fahimirad S., Fahimirad Z., Sillanpää M. (2021). Efficient removal of water bacteria and viruses using electrospun nano-fibers. J. Sci. Total Environ..

[9] Roche R., Yalcinkaya F. (2018). Incorporation of PVDF Nanofibre Multilayers into Functional Structure for Filtration Applications. Nanomaterials.

[10] Liu P., Zhu C., Mathew A.P. (2019). Mechanically robust high flux graphene oxide—Nanocellulose membranes for dye removal from water. J. Hazard. Mater..

[11] Mataram A., Ismail A.F., Yuliwati E., Matsuura T., Zamheri A., Rizal S. (2015). Water Treatment Perfomance: Application of Electrospun Nanofibers. J. Teknol..

[12] Li X., Liu T., Zhang Y., Cai J., He M., Li M., Chen Z., Zhang L. (2020). Growth of BiOBr/ZIF-67 Nanocomposites on Carbon Fiber Cloth as Filter-Membrane-Shaped Photocatalyst for Degrading Pollutants in Flowing Wastewater. Adv. Fiber Mater..

[13] Li S., Cai M., Liu Y., Wang C., Lv K., Chen X. (2022). S-Scheme photocatalyst TaON/Bi2WO6 nanofibers with oxygen vacancies for efficient abatement of antibiotics and Cr(VI): Intermediate eco-toxicity analysis and mechanistic insights. Chin. J. Catal..

[14] Koley G. (2019). Editorial for the Special Issue on MEMS/NEMS Sensors: Fabrication and Application. J. Micromachines.

[15] Islam M.S., Ang B.C., Andriyana A., Afifi A.M. (2019). A review on fabrication of nanofibers via electrospinning and their applications. SN Appl. Sci..

[16] Hu J., Zhang K.-Q. (2019). Electrospun Nanofibers for Optical Applications. Electrospinning: Nanofabrication and Applications.

[17] Haider A., Haider S., Kang I.-K. (2018). A comprehensive review summarizing the effect of electrospinning parameters and potential applications of nanofibers in biomedical and biotechnology. Arab. J. Chem..

[18] Ibrahim H.M., Klingner A. (2020). A review on electrospun polymeric nanofibers: Production parameters and potential applications. Polym. Test..

[19] Pillay V., Dott C., Choonara Y.E., Tyagi C., Tomar L., Kumar P., Du Toit L.C., Ndesendo V.M. (2013). A review of the effect of processing variables on the fabrication of Electrospun nanofibers for drug delivery applications. J. Nanomater..

[20] Amna R., Ali K., Malik M.I., Shamsah S.I. (2020). A Brief Review of Electrospinning of Polymer Nanofibers: History and Main Applications. J. New Mater. Electrochem. Syst..

[21] Huang L., Arena J.T., Manickam S.S., Jiang X., Willis B.G., McCutcheon J.R. (2014). Improved mechanical properties and hydrophilicity of electrospun nanofiber membranes for filtration applications by dopamine modification. J. Membr. Sci..

[22] Abd Halim N.S., Wirzal M.D., Bilad M.R., Md Nordin N.A., Adi Putra Z., Sambudi N.S., Mohd Yusoff A.R. (2019). Improving performance of Electrospun nylon 6,6 Nanofiber membrane for produced water filtration via solvent vapor treatment. J. Polym..

[23] Guibo Y., Qing Z., Yahong Z., Yin Y., Yumin Y. (2012). The electrospun polyamide 6 nanofiber membranes used as high efficiency filter materials: Filtration potential, thermal treatment, and their continuous production. J. Appl. Polym. Sci..

[24] Pereao O.K., Bode-Aluko C., Ndayambaje G., Fatoba O., Petrik L.F. (2016). Electrospinning: Polymer Nanofibre Adsorbent Applications for Metal Ion Removal. J. Polym. Environ..

[25] Rianjanu A., Kusumaatmaja A., Suyono E.A., Triyana K. (2018). Solvent vapor treatment improves mechanical strength of electrospun polyvinyl alcohol nanofibers. J. Heliyon.

[26] Munaweera K.I., Kottegoda N. (2022). A comprehensive review on electrospun nanohybrid membranes for wastewater treatment. Beilstein J. Nanotechnol..

[27] Okutan N., Terzi P., Altay F. (2022). Affecting parameters on electrospinning process and characterization of electrospun gelatin nanofibers. Food Hydrocoll..

[28] Fauzi A., Hapidin D.A., Munir M.M., Iskandar F., Khairurrijal K. (2020). A superhydrophilic bilayer structure of a nylon 6 nanofiber/cellulose membrane and its characterization as potential water filtration media. RSC Adv..

[29] Huang L., Manickam S.S., McCutcheon J.R. (2013). Increasing strength of electrospun nanofiber membranes for water filtration using solvent vapor. J. Membr. Sci..

[30] Liu C., Li X., Liu T., Liu Z., Li N., Zhang Y., Xiao C., Feng X. (2016). Microporous CA/PVDF membranes based on electrospun nanofibers with controlled crosslinking induced by solvent vapor. J. Membr. Sci..

[31] Li H., Zhu C., Xue J., Ke Q., Xia Y. (2017). Enhancing the Mechanical Properties of Electrospun Nanofiber Mats through Controllable Welding at the Cross Points. Macromol. Rapid Commun..

[32] Wu H., Shi J., Ning X., Long Y.-Z., Zheng J. (2022). The High Flux of Superhydrophilic-Superhydrophobic Janus Membrane of cPVA-PVDF/PMMA/GO by Layer-by-Layer Electrospinning for High Efficiency Oil-Water Separation. J. Polym..

[33] El-Aswar E.I., Ramadan H., Elkik H., Taha A.G. (2022). A comprehensive review on preparation, functionalization and recent applications of nanofiber membranes in wastewater treatment. J. Environ. Manag..

[34] Attari N., Hausler R. (2022). Mechanical Characterization of Nanocelluloses/Cellulose Acetate Composite Nanofibrous Membranes. Res. Sq..

[35] Namsaeng J., Punyodom W., Worajittiphon P. (2019). Synergistic effect of welding electrospun fibers and MWCNT reinforcement on strength enhancement of PAN–PVC non-woven mats for water filtration. J. Chem. Eng. Sci..

[36] Khatri M., Francis L., Hilal N. (2023). Modified Electrospun Membranes Using Different Nanomaterials for Membrane Distillation. Membranes.

[37] Ehrmann A. (2021). Non-Toxic Crosslinking of Electrospun Gelatin Nanofibers for Tissue Engineering and Biomedicine—A Review. Polymers.

[38] Alias A.H., Norizan M.N., Sabaruddin F.A., Asyraf M.R., Norrrahim M.N., Ilyas A.R., Kuzmin A.M., Rayung M., Shazleen S.S., Nazrin A. (2021). Hybridization of MMT/Lignocellulosic Fiber Reinforced Polymer Nanocomposites for Structural Applications: A Review. Coatings.

[39] Covelo A., Rodil S., López-Villegas E.O., Álvarez C.A., Hernandez M. (2020). Evaluation and correlation of electrochemical and mechanical properties of PVA/SA nanofibres. Surf. Interface Anal..

[40] Jeong L., Lee K.Y., Liu J.W., Park W.H. (2006). Time-resolved structural investigation of regenerated silk fibroin nanofibers treated with solvent vapor. Int. J. Biol. Macromol..

[41] Cai J., Zhang Q., Lei M., He J., Liu G. (2016). The use of solvent-soaking treatment to enhance the anisotropic mechanical properties of electrospun nanofiber membranes for water filtration. RSC Adv..

[42] Wang Y., Górecki R.P., Stamate E., Norrman K., Aili D., Zuo M., Guo W., Hélix-Nielsen C., Zhang W. (2019). Preparation of super-hydrophilic polyphenylsulfone nanofiber membranes for water treatment. RSC Adv..

[43] Kiani S., Mousavi S., Shahtahmassebi N., Saljoughi E. (2015). Preparation and characterization of polyphenylsulfone nanofibrous membranes for the potential use in liquid filtration. J. Desalination Water Treat..

[44] Ozbey-Unal B., Gezmis-Yavuz E., Eryildiz B., Koseoglu-Imer D.Y., Keskinler B., Koyuncu I. (2020). Boron removal from geothermal water by nanofiber-based membrane distillation membranes with significantly improved surface hydrophobicity. J. Environ. Chem. Eng..

[45] Li L., Hashaikeh R., Arafat H.A. (2013). Development of eco-efficient micro-porous membranes via electrospinning and annealing of poly (lactic acid). J. Membr. Sci..

[46] Guclu S., Pasaoglu M.E., Koyuncu I. (2015). Membrane manufacturing via simultaneous electrospinning of PAN and PSU solutions. Desalination Water Treat..

[47] Homaeigohar S., Koll J., Lilleodden E.T., Elbahri M. (2012). The solvent induced interfiber adhesion and its influence on the mechanical and filtration properties of polyethersulfone electrospun nanofibrous microfiltration membranes. Sep. Purif. Technol..

[48] Sallakhniknezhad R., Khorsi M., Niknejad A.S., Bazgir S., Kargari A., Sazegar M., Rasouli M., Chae S. (2021). Enhancement of physical characteristics of styrene–acrylonitrile Nanofiber membranes using various post-treatments for membrane distillation. J. Membr..

[49] Nauman S., Lubineau G., Alharbi H.F. (2021). Post Processing Strategies for the Enhancement of Mechanical Properties of ENMs (Electrospun Nanofibrous Membranes): A Review. J. Membr..

[50] Diantoro M., Kusumaatmaja A., Triyana K. (2018). Stabilization of PVA/Chitosan/TiO2 Nanofiber Membrane with Heat Treatment and Glutaraldehyde Crosslink. IOP Conf. Ser. Mater. Sci. Eng..

[51] Tonglairoum P., Sutananta W., Rojanarata T., Ngawhirunpat T., Opanasopit P. (2014). Thermally Crosslinked Chitosan-EDTA/PVA Electrospun Nanofiber Mats: Crosslinking Conditions. J. Adv. Mater. Res..

[52] Li Y., Shen Q., Shen J., Ding X., Liu T., He J., Zhu C., Zhao D., Zhu J. (2021). Multifunctional fibroblasts enhanced via thermal and freeze-drying post-treatments of aligned Electrospun Nanofiber membranes. J. Adv. Fiber Mater..

[53] Li M., Sheng L., Zhang H., Yang Y., Xu R., Bai Y., Song S., Liu G., Wang T., Huang X. (2020). Effect of the heat treatment temperature on mechanical and electrochemical properties of polyimide separator for lithium ion batteries. J. Mater. Sci..

[54] Zhang C., Liang Y., Yao L., Qiu Y. (2015). Effect of thermal treatment on the properties of electrospun LiFePO_4_–carbon nanofiber composite cathode materials for lithium-ion batteries. J. Alloys Compd..

[55] Su C., Lu C., Cao H., Tang K., Chang J., Duan F., Ma X., Li Y. (2018). Fabrication and post-treatment of nanofibers-covered hollow fiber membranes for membrane distillation. J. Membr. Sci..

[56] Wongkrongsak S., Pangon A., Pongsak N., Piroonpan T., Pasanphan W. (2022). Strengthened Silk-Fibroin/Poly(ethylene oxide) Nonwoven Nanofibers: A Dual Green Process Using Pure Water for Electrospinning and Electron Beam-Assisted Cross-Linking. ACS Sustain. Chem. Eng..

[57] Prabhu N.N., Jagadeesh Chandra R.B., Rajendra B.V., George G., Mourad A.H.I., Shivamurthy B. (2022). Electrospun Zinc Oxide Nanofiber Based Resistive Gas/Vapor Sensors—A Review. Eng. Sci..

[58] Yusof M.R., Shamsudin R., Zakaria S., Hamid M.A.A., Yalcinkaya F., Abdullah Y., Yacob N. (2020). Electron-Beam Irradiation of the PLLA/CMS/β-TCP Composite Nanofibers Obtained by Electrospinning. Polymers.

[59] Ray S.S., Bakshi H.S., Dangayach R., Singh R., Deb C.K., Ganesapillai M., Chen S.-S., Purkait M.K. (2020). Recent developments in nanomaterials-modified membranes for improved membrane distillation performance. Membranes.

[60] Domaschke S., Zündel M., Mazza E., Ehret A.E. (2019). A 3D computational model of electrospun networks and its application to inform a reduced modelling approach. Int. J. Solids Struct..

[61] Chavoshnejad P., Alsmairat O.Q., Ke C., Razavi M.J. (2021). Effect of interfiber bonding on the rupture of electrospun fibrous mats. J. Phys. D Appl. Phys..

[62] Yin Y., Xiong J. (2018). Finite element analysis of electrospun nanofibrous mats under biaxial tension. Nanomaterials.

[63] Barhoum A., Pal K., Rahier H., Uludag H., Kim I.S., Bechelany M. (2019). Nanofibers as new-generation materials: From spinning and nano-spinning fabrication techniques to emerging applications. Appl. Mater. Today.

[64] Fadil F., Affandi N.D.N., Misnon M.I., Bonnia N.N., Harun A.M., Alam M.K. (2021). Review on electrospun nanofiber-applied products. Polymers.

[65] Chen H., Huang M., Liu Y., Meng L., Ma M. (2020). Functionalized electrospun nanofiber membranes for water treatment: A review. Sci. Total Environ..

